# Supplementary material for: Are carbon emissions trading and green financial instruments synergistic? -Comprehensive quantitative research based on content analysis
Source: PLoS One. 2024 Mar 7;19(3):e0298601. doi: 10.1371/journal.pone.0298601 (PMC10919601; doi:10.1371/journal.pone.0298601)
Supplement: S1 Data — (ZIP) [file pone.0298601.s001.zip › Data/地级市Z_F工作报告.zip!/╣π╢1⁄2/╣π╓▌╩╨/└┤╫╘╣1⁄2╓┌║┼í╛┬φ┐╦╩2╛▌═°í┐_2010.docx]

各位代表：

　　我代表广州市人民政府向大会作政府工作报告，请予审议，并请政协委员和其他列席人员提出意见。

　　一、2009年工作回顾

　　2009年是新世纪以来我市经济发展最为困难的一年。在省委、省政府和市委的正确领导下，我们坚持以邓小平理论和“三个代表”重要思想为指导，以科学发展观统揽全局，认真贯彻落实中央和省一系列应对国际金融危机、促进经济平稳较快发展的决策部署，积极实施《珠江三角洲地区改革发展规划纲要》（以下简称《规划纲要》），扎实推进“三促进一保持”，团结一心，攻坚克难，全力保增长、保民生、保稳定，各项工作取得一定成效，经济社会发展好于预期。

　　（一）保增长成效显著。面对国际金融危机的严峻形势，我们始终把保增长作为首要任务，积极实施扩大内需、促进发展的政策措施，推动经济平稳较快增长。去年，全市地区生产总值9113亿元，增长11.5％，比预期目标高1.5个百分点；人均地区生产总值88834元，增长10％。源于广州地区的财政一般预算收入2656亿元，增长7.2％；地方财政一般预算收入703亿元，增长13％。投资增长势头强劲，内需成为拉动经济增长的主要动力。全社会固定资产投资2660亿元，增长22.3％，其中81个重点项目完成投资946亿元，占全社会固定资产投资的35.6％。大力开拓国内市场，增强消费需求对经济增长的拉动作用。全年实现社会消费品零售总额3648亿元，增长16.2％。努力克服外需不足的困难，创新外贸发展方式，外贸进出口降幅逐步收窄。全年进出口总值767亿美元，下降6.4％，其中出口总值374亿美元，下降13％。利用外资稳定增长，全市实际使用外商直接投资38亿美元，增长4.2％，批准投资总额超1000万美元以上的大项目145个。

　　（二）调结构取得重要成果。坚持解决当前困难与促进长远发展相结合，紧紧抓住国际金融危机形成的倒逼机制，积极主动调整结构，促进产业转型升级，使应对国际金融危机的过程成为调结构、促转变的过程。年末，三次产业比重为1.9︰37.2︰60.9。出台了《广州市建设现代产业体系规划纲要》，重点推进五大现代服务业功能区规划建设，促进现代服务业加快发展。全市第三产业实现增加值5546亿元，增长13.6％，对经济增长贡献率达70.1％。金融业实现增加值553亿元，增长29％，创近年来新高。物流业发展能力进一步增强，白云国际机场年旅客吞吐量3705万人次，增长10.8％，货邮吞吐量增幅达31.3％。广州港货物吞吐量3.64亿吨，增长4.9％，其中集装箱吞吐量达1120万标箱，增长1.8％，列全国第三位。出台了《关于加快会展业发展的若干意见》，广交会、广博会、留交会、国际汽车展、国际设计周等品牌会展影响力不断提升。出台了《关于加快我市旅游业发展建设旅游强市的意见》，旅游产品体系建设初见成效，全市旅游业总收入994亿元，增长18.7％。软件和信息服务业收入达1150亿元，增长21％。

文化及相关产业增加值占全市GDP的7.87％。先进制造业进一步发展壮大。汽车、石化、造船、数控装备、电子信息等先进制造业基地和国家数字家庭应用示范产业基地建设加快推进，与中央企业及国内大型企业战略合作取得积极进展，制造业加速向高端化、集群化转型升级。全年完成工业总产值13481亿元，增长9.9％。规模以上工业企业利润增长22.4％。三大支柱产业完成产值5243亿元，增长20.3％。汽车制造业逆势而上，产值增长30.4％，年产量达113万辆。龙穴造船基地30.8万吨超大型油轮下水和东方电气生产出我国首台国产化百万千瓦级核电压力容器，标志着我市工业制造能力实现了新突破。高新技术产业加快发展。全市规模以上工业高新技术产品产值4104亿元，增长17.3％，占工业总产值比重达32.8％。自主创新体系建设取得重大进展。我市被列为国家创新型试点城市，广州高新区成为全国首批国家级创新型科技园区和国家海外高层次人才创新创业基地。中新知识城建设前期工作及商务谈判进展顺利，为签约奠定了良好基础。知识产权创造能力进一步提升，全市专利申请量增长18.2％，其中发明专利申请量增长24.6％，发明专利授权量增长35.1％。中小企业和民营经济稳步发展。取消119项行政事业性收费，减轻企业负担25亿元。加大对中小企业信用担保体系建设的扶持力度，出台一系列政策措施，帮助企业克服困难。全市民营经济实现增加值3164亿元，增长12.5％，占GDP的比重达34.7％。“双转移”和中心城区产业“退二进三”步伐加快。去年共向市外转移企业61家，停产、关闭或搬迁“退二”企业95家，淘汰落后企业270多家，利用旧厂房（仓库）发展现代服务业项目60多个。全市万元生产总值能耗下降4％以上，二氧化硫和化学需氧量排放量分别下降8％以上和6.2％以上。

　　（三）贯彻实施《规划纲要》先行先试工作开局良好。邀请国家发改委宏观经济研究院围绕国家中心城市的战略定位和关系广州科学发展的若干重要问题进行研究。组织编制了贯彻《规划纲要》实施细则，开展了主体功能区规划、新一轮城乡总体规划和土地利用总体规划的修编工作，并加快推进控制性详细规划城乡全覆盖工作。以广佛同城化为突破口推进珠三角一体化发展迈出实质性步伐，先后与佛山、肇庆、江门、云浮等市签署了合作框架协议和9个专项合作协议，完善了区域协调联动机制；广佛同城化建设52项和广佛肇经济圈37项重点工作全面展开。成功举办穗港现代服务业合作交流会，深化实施CEPA及服务业先行先试工作，不断推进穗港澳更紧密合作。制定实施《关于进一步推动国有企业改革与发展的意见》及其配套文件，取消65个国有企业行政级别，初步建立起以产权为纽带的国有企业领导人分类分级管理体制，进一步健全了公司治理机制。广州银行和广州农村商业银行改革重组和改制取得历史性突破，成功解决了历史遗留问题，化解了潜在风险，实现了新的发展。广州开发区创新发展模式和增城市统筹城乡综合配套改革试点工作扎实推进。城市建设投融资体制改革进一步深化，城建和水务集团在综合整治人居环境和水环境中发挥了重要作用。制定实施《关于学习借鉴香港先进经验推进社会管理改革先行先试的意见》，积极探索社会管理体制改革，在社区服务、社会工作人才队伍建设和政府购买服务等方面取得初步成效。启动了医药卫生体制改革。文化体制改革继续深化，荣获“全国文化体制改革先进地区”称号，促进了文化事业和文化产业协调发展。

　　（四）亚运城市行动计划全面推进。亚运场馆建设和开、闭幕式筹备工作按期推进，建成我国内地首个“无规定马属动物疫病区”，赛时交通、住宿、餐饮、医疗卫生、安全保卫等服务保障准备工作以及市场开发、宣传推广活动有序开展。亚运城建设探索出“政府主导，成片开发，完善配套，整体出让”的新模式，体现了赛时服务与赛后利用的有机结合，拉开了城市发展空间布局，成为我市大型社区开发建设的典范。围绕实现“天更蓝、水更清、路更畅、房更靓、城更美”的目标，全力实施2010年城市环境面貌“大变”工程。白云国际机场、广州港扩建和续建工程加快推进，广州南站、武广高速铁路建成投入使用。总里程32公里的地铁五号线建成通车，全市轨道交通通车里程达150公里。一批高速公路和城市快速路抓紧建设。在公交优先方面进行了实质性探索，全程22.9公里的快速公交（BRT）试验线投入运营。新增停车泊位5.86万个，完成年度计划117％。继续推动实施“青山绿地”、“蓝天碧水”二期工程，城市公园、森林公园和自然保护区的建设保护进一步加强。全市森林覆盖率达38.2％，林木绿化率44.8％，新增32个公园免费对市民开放。加快推进天然气置换工作，中心城区管网已全部置换，全市新增天然气用户36.24万户。大力推进迎亚运人居环境综合整治工程，对全市81条主干道、74个亚运场馆周边地区以及153个城市社区和片区进行人居环境综合整治。完成了城市各主要出入口景观绿化升级工程。制定了旧城区和“城中村”改造规划和政策措施，改造工作加紧推进。克服重重困难，开展我市有史以来最大规模的污水治理和河涌综合整治工程，重点整治121条河涌，建设38座生活污水处理厂和覆盖全市32个镇（街）245个村的农村生活污水处理设施，铺设1140公里长的截污管网，建设白云湖、海珠湖等调水补水工程，并对全市228处水浸街进行改造。581项治水工程全面铺开，过半项目已经完工，特别是影响和制约工程进度的征地拆迁任务基本完成，为全面完成整治任务奠定了坚实基础。西江引水工程按计划推进，已完成管网铺设30公里。全力推进“空气整治50条”措施的落实，开展工业企业降氮脱硝、清洁能源改造、挥发性有机物排放控制、饮食服务业污染整治和扬尘污染控制工作。加大环保执法力度，查处违法案件3769宗，关停不符合环保要求的企业或项目1546个，解决2万多件污染扰民问题。实施机动车环保标志管理，对全市20多万辆黄标车实行限行措施。积极推动珠三角城市群空气污染联防联治，空气环境质量不断改善。去年，全市空气质量优良率为95.1％，同比上升0.8个百分点；二氧化硫、二氧化氮、可吸入颗粒物平均浓度均继续优于国家环境空气质量二级标准。大力整顿和规范市场经济秩序，不断完善产品、食品、药品质量安全长效监督机制。强化城市综合执法，依法加大对“六乱”的整治力度，清除违法户外广告面积7.3万平方米，制止和清拆违法建设面积132万平方米。

　　（五）民生福利继续改善。认真落实市委、市政府66条惠民措施和17条补充意见，努力保障和改善民生。去年，市本级一般预算安排用于民生和各项社会事业的资金220.5亿元，占财政支出73.6％，比上年提高3.9个百分点。实施积极的就业政策，全市城镇新增就业人数72.45万人，占全省的2/5和全国的1/15，其中本市城镇户籍新增就业人数34.38万人，增长34％。失业人员就业率达70.07％，上升2.17个百分点；登记失业率2.25％，下降0.07个百分点。社会保障水平有新的提高。全市社会保险参保达1856万人次，增加206万人次，增长12％。企业退休人员月人均养老金增加510元，达到2057元，增幅达33％。通过阶段性调低缴费比例和缴费基数，累计为企业和参保个人减轻负担53.4亿元。农村最低生活保障标准由每人每月平均220元提高到271元，增幅为23％，提前3年实现了农村低保标准为城市70％以上的目标。各类优抚对象抚恤补助标准平均增幅15.1％。农转居人员基本养老金上调至每人每月521元，增长22％；失业保险金提高到每月791元，提高了15％。制订实施外来从业人员基本医疗保险办法，养老、失业和工伤保险市级统筹办法和解决非本市户籍人员领取基本养老待遇问题的办法。医疗救助覆盖城乡居民，共资助5.2万名困难群众参加城镇居民基本医疗保险和新型农村合作医疗，为12.31万人次减免了医疗费用。改建、重建了3310户农村助残安居房。慈善事业不断发展，确定每年12月12日为“广州慈善日”。居家养老、老年人优待等社会福利服务体系进一步完善，为84.8万老年人发放了集多种功能于一体的社保卡。住房保障水平持续提高，全市新开工保障性住房项目14个，建筑面积186万平方米。为1.77万户低收入家庭解决了住房困难问题，累计解决了3.99万户低收入家庭的住房困难问题，完成总目标任务的51.72％。建立了职工工资水平与企业经济效益协调增长机制，城市居民年人均可支配收入27610元，增长9.1％。

　　（六）统筹城乡发展力度加大。制定实施《关于加快形成城乡经济社会发展一体化新格局的实施意见》及12个配套文件，大力统筹城乡经济社会发展。全市实现农业总产值296亿元，增长4.2％。农村居民人均纯收入11067元，增长12.6％，连续两年高于城市居民收入增速。完成18宗城乡水利防灾减灾和近14万亩农田、1.9万亩鱼塘的标准化建设任务，规划建设了30个都市型现代农业示范区，市级以上农业龙头企业达到80家。扶持发展区（县级市）购物中心、大型批发市场和连锁网点118个，近20万户农民受益。农村改革取得实质性进展，成立了广州农村产权交易所，全市农村土地承包经营权流转面积达54.05万亩，占家庭承包经营耕地总面积的34.1％。农村劳动力培训转移就业力度加大，全年培训农村劳动力5.45万人，转移就业7.15万人。乡村旅游蓬勃发展，扶持培育了一批特色乡村旅游品牌景点。新农村建设扎实推进，完成1139个行政村规划编制，累计对774个行政村进行村容村貌整治。继续推进中心镇建设，加大对北部山区镇的扶持力度。农村社保工作全面铺开，参加农村社会养老保险的农村居民达33万人。农村社会事业持续进步，农民享受的公共服务日益增多，保障水平逐步提高。

　　（七）社会事业稳步发展。进一步巩固教育优先发展地位，市本级财政对教育投入占一般预算支出达18.2％。市、区两级共投入5.3亿元，实施免费义务教育，惠及79万名学生。义务教育规范化学校建设加快推进，普通高中优质发展，省市一级学校提供的学位占全市普通高中的89.1％，高考总体成绩创20年来新高。中等职业教育品牌建设得到加强，我市中职学生在全国中职技能大赛中名列全国省会城市总分第一名。市属高校强化内涵发展，综合实力进一步提升。落实教师待遇，解决了全市2246名代课教师问题。公共文化服务体系建设不断完善，城乡基层文化设施基本实现全覆盖。文化遗产保护工作取得新进展，粤剧被联合国教科文组织列入“人类非物质文化遗产代表作名录”。城乡体育设施不断完善，群众体育活动广泛开展。竞技体育人才辈出，广州运动员参加第十一届全运会创造历史最好成绩。成功举办苏迪曼杯羽毛球赛、女子世乒赛、广州国际女子网球公开赛、亚洲田径锦标赛等54项国内外重大赛事。重大疾病防控和突发公共卫生事件应急处置工作继续加强，甲型H1N1流感等重大传染病得到有效防控。社区卫生服务机构建设有效推进，完成市妇女儿童医疗中心等一批医疗卫生建设项目。农村三级医疗卫生服务体系不断健全，农村合作医疗参合率达99.7％。实施“健康亚运、健康广州”全民健康活动。开展“争当好市民、当好东道主——亚运广州行”等系列活动，推进新一轮创建全国文明城市工作，公共文明指数测评成绩位居全国同类城市前列。国际交流与合作成绩显著，成功举办2009年世界城市和地方政府联合组织（UCLG）世界理事会会议暨广州国际友城大会，广州的国际声誉和影响力进一步提高。“信息广州”建设取得新进展，电子政务便民服务成效显著，政府门户网站绩效居全国前列。对口帮扶工作有序展开。全年招收358名梅州农村贫困家庭学生入读市属公办中等职业学校。加快对口支援汶川威州镇恢复重建工作，投入资金28亿元，77项援建项目全面完成并交付使用，提前半年实现了中央和省关于“三年援建任务两年基本完成”的目标。第二次全国经济普查取得阶段性成果。低生育水平进一步稳固，人口和计生利益导向政策体系逐步完善，初步解决了独生子女父母退休时一次性奖励兑现问题，受惠群众逾10万人。“六好”平安和谐社区建设深入开展，58.6％的社区成为全省“六好”平安和谐社区。流动人员和出租屋管理进一步加强，“房中房”整治取得明显成效。社会治安形势明显好转，全市刑事案件发案数下降12.8％，其中，抢劫、抢夺案件同比分别下降21.4％和24.2％。安全生产形势继续保持稳定，全市事故起数、死亡人数同比分别下降17.4％和9.1％。“扫黄打非”、知识产权保护和打击走私成效明显。国防动员、国防后备力量建设、人民防空、海防建设以及优抚安置工作扎实推进，拥军优属、军民共建活动深入开展。侨务工作走在全省前列。民族、宗教、对台事务、档案、气象、参事、妇女、儿童、残疾人事业取得新进步，市政府再次被国务院评为全国民族团结进步模范集体。

　　（八）民主法制和政府自身建设不断加强。坚持定期向人大报告工作，认真执行市人大及其常委会决议，自觉接受监督。积极改进人大代表建议、批评和意见及政协提案的办理工作，全年办理答复927件，办理质量和效率进一步提高。主动接受政协的民主监督，加强与各民主党派、工商联、工会、共青团、妇联的联系，落实市政府各职能部门与各民主党派和工商联对口联系制度。充分听取社会各界的意见和建议，科学化、民主化决策机制进一步完善。进一步加强政府自身建设。完成了新一轮政府机构改革和行政审批制度改革，在9个部门实行大部制，市政府工作部门和办事机构从49个精简为40个，行政审批事项减少559项，精简66.2％。制定了《广州市全面推进依法行政建设法治政府五年规划》和《广州市规范行政执法自由裁量权规定》，加快法治政府建设，规范行政执法行为。加强公务员队伍教育和管理，公务员整体素质和服务水平有新的提高。市政务服务中心功能不断完善，政务公开、新闻发布、重大事项公示和听证制度得到进一步落实，率先在全国公布政府部门预算。加强财政支出绩效评价，严格控制党政机关一般性行政经费支出，实现了公务购车和用车经费、会议经费、公务接待费用、党政机关出国（境）费用以及办公经费预算“五个零增长”。坚持不懈地开展廉政建设和反腐败斗争，切实加强执法、廉政和效能监察。纠风治乱专项工作深入开展。实施《广州市党政领导干部问责暂行办法》和《广州市领导干部任期经济责任审计实施办法》，推进行政问责、审计工作，政务环境不断优化。

　　各位代表，过去一年，我们在应对国际金融危机的重大考验中，取得来之不易的成绩，也积累了极为宝贵的经验。这是全市人民和社会各界团结一心、共同奋斗的结果。在此，我谨代表广州市人民政府，向全市人民、驻穗解放军和武警部队官兵、各民主党派、人民团体以及社会各界人士表示崇高的敬意!向所有关心和支持广州现代化建设的港澳同胞、台湾同胞、海外侨胞和国际友人表示衷心的感谢！

　　在充分肯定成绩的同时，我们也十分清醒地看到，我市经济社会发展仍然存在不少问题和薄弱环节：受国际金融危机影响，外需不足的状况没有根本改变，部分企业生产经营还很困难，影响经济平稳较快增长的不稳定因素依然存在；经济发展面临的人口、资源和环境压力很大，转变发展方式和调整结构的任务十分紧迫；城市功能、公共服务、自主创新能力等与国家中心城市要求仍有不小差距；城乡和地区间发展还不平衡，宜居城乡“首善之区”建设任重道远；保障和改善民生还有许多工作要做，仍需作出更大努力；城市管理的科学化、精细化水平不够高，部分地区交通拥堵问题比较突出，离人民群众的要求还有差距。我们要正视这些问题，采取更加有效的措施破解难题，把各项工作做得更好。

　　二、2010年工作安排

　　今年是广州发展史上极其重要的一年。我们既要承载全国人民和亚洲人民的重托，全力以赴办好亚运会和亚残运会，提升广州的国际影响力，又要保持经济平稳较快发展，全面完成“十一五”规划，推动科学发展向全面深化转变。做好今年工作，关系当前，影响长远，意义十分重大。我们要充分认识面临形势和任务的复杂性、艰巨性，进一步增强忧患意识和责任意识，切实把思想和行动统一到党中央、国务院和省委、省政府对形势的科学判断和决策部署上来，坚定信心，知难而进，推动经济社会又好又快发展。

　　今年政府工作的总体要求是：以邓小平理论和“三个代表”重要思想为指导，深入贯彻落实科学发展观，认真落实党的十七大及十七届四中全会、中央经济工作会议和省委十届六次、市委九届八次全会精神，紧紧围绕加快建设国家中心城市、全面提升科学发展实力的奋斗目标，坚持一手抓经济社会发展，一手抓亚运筹办工作，坚定不移调结构，脚踏实地促转变，着力推进“三促进一保持”，着力加强城市环境建设，着力深化改革开放，着力统筹城乡发展，着力改善民生福利，全面完成“十一五”规划确定的各项目标任务，确保实现城市环境面貌“大变”目标，圆满成功举办广州亚运会和亚残运会，实现“迎接亚运会，创造新生活”的要求。

　　今年经济社会发展主要预期目标是：地区生产总值增长10％；人均生产总值增长8.8％；全社会固定资产投资增长15％；社会消费品零售总额增长13％；外贸出口总值增长5％；万元生产总值能耗下降4.2％，二氧化硫和化学需氧量排放量分别下降7.5％和4％；城市居民人均可支配收入增长10％；农村居民人均纯收入增长10％；居民消费价格总水平涨幅控制在3％左右；城镇登记失业率控制在3.5％以内；人口自然增长率控制在6.5％。以内。重点抓好以下九项工作：

　　（一） 举全市之力做好亚运筹办工作，确保亚运会和亚残运会圆满成功。

　　认真做好场馆建设、赛事组织和服务保障工作。按时保质完成亚运城、亚运场馆及相关配套设施建设。整合政府和社会资源，组织亚运场馆运行团队，建立赛时运行指挥体系，组织好测试赛、火炬传递、开闭幕式等重要活动。广泛传播“激情盛会、和谐亚洲”理念，激发亚洲各国和地区公众及媒体关注、参与、支持广州亚运会的热情。深入开展“迎亚运、讲文明、树新风、促和谐”活动，增强市民当好东道主意识，弘扬讲文明、重礼仪、团结友善、热情好客的良好风尚，尊重各个国家、地区各民族的风俗习惯和宗教文化信仰，充分展示广州文明形象。发挥驻穗中央和省属单位、部队等各方面参与和支持亚运的积极性，促进共驻共建共同发展。以“一流的组织管理、一流的场馆设施、一流的城市环境、一流的服务水平、一流的社会风尚”迎接亚运会和亚残运会，实现两个亚运“同时举办，同样精彩”。

　　确保实现城市环境“大变”目标。以大干促大变精神，继续实施“青山绿地”、“蓝天碧水”二期工程和花园城市行动纲要，全面推进城市环境综合整治工作，使广州“天更蓝、水更清、路更畅、房更靓、城更美”。大力推进空港、海港和铁路等重大枢纽型交通基础设施建设。加快东新、南二环、广深沿江广州段等高快速路建设。加快发展轨道交通，确保亚运会举办时地铁三四号线北延段、二八号线延长线及广佛线首段建成开通，全市轨道交通开通里程达到218公里，线网覆盖80％的亚运比赛场馆。高标准抓好城市绿化美化工作，推进城乡森林体系、园林绿地体系、田园景观体系建设，鼓励企业和个人认捐、认种树木，增加森林碳汇，构建广州绿色生态屏障。加快落实“空气整治50条”及“新31条”污染综合治理措施，密切与周边城市的环境保护合作，采取更加严格的控制手段，加强对环境质量的监控。重点抓好工业企业、机动车、饮食企业、工地扬尘等七大类污染的综合整治，确保亚运会期间全市空气质量符合国家标准和国际基本要求。再接再厉，毫不松懈地抓好污水治理和河涌综合整治，同步做好截污、清淤、补水和两岸景观绿化工作。全面清拆饮用水源保护区内违法排污口，从严查处向江河湖泊直接排放污水的行为，确保亚运前实现水环境质量明显好转。全面推进人居环境综合整治，完成城市主干道、高架路沿线两侧建筑立面，亚运场馆与接待宾馆周边地区整饰工作，推进整治工作向社区深化延伸。以推进垃圾分类为突破口，提高垃圾减量化、资源化和无害化处理水平。加快推进“三旧”改造，围绕力争用3~5年时间基本完成全市重点区域成片改造和10年内基本完成138条“城中村”改造的目标，今年启动10个片区的旧城区改造，力争完成9条“城中村”拆除任务。改造时，坚持保护历史文化与改善环境相结合，严格执行“三条线”控制要求：“绝对保护线”内建筑不得拆除，必须严格按原状予以维修，传承历史文化风貌;“重点保护线”内建筑按“修旧如旧、建新如故”原则进行改造;“更新改造线”内建筑可实施成片拆除改造。

　　切实保障城市安全和谐稳定。加大城市管理工作力度，理顺城市管理体制，大力推进综合执法重心下移和数字城管建设，推广城市区域式管理和综合巡检，进一步提高城市管理科学化、精细化水平。规范市场经济秩序，强化产品、食品、药品质量安全监管，做好亚运食品药品安全保障工作。加强疫情监控，推进安全生产管理监督和源头治理，积极防控重大公共卫生事件，坚决杜绝重大安全生产事故。加强亚运应急预案演练，着力提高赛时应对突发事件、处置复杂问题的能力，确保赛事期间安全有序、万无一失。抓好维护社会大局稳定和矛盾纠纷排查化解工作，强化社会治安综合治理，创新群防群治工作机制，加强出租屋管理和各类人员密集场所的安全检查，有效预防和依法严厉打击各类违法犯罪活动，确保实现“平安亚运”。

　　（二）加快经济发展方式转变和结构调整，不断提高经济发展质量和效益。

　　以促进高端发展和绿色增长为取向调整优化产业结构和布局。大力构建现代产业体系，加快发展低碳经济，促进产业转型升级。中心城区要依托五大现代服务业功能区，大力发展以现代服务业为主导的服务经济，强化国家中心城市功能。把珠江新城-员村地区建设成为国际金融商务核心区，琶洲地区建设成为国际会展商务区，城市新中轴线南段地区建设成为行政办公中心，白鹅潭地区建设成为国际商业中心，白云新城地区建设成为宜居新城及商业文化服务中心。加快推进科学城金融创新服务区、南沙保税港区和广州空港保税物流中心建设，规划建设“广州北岸文化码头”创意产业园区，大力推进广州国际商品展贸城、国际医药港及大宗商品电子交易中心建设，支持建设越秀区北部核心产业功能提升区和北京路广府文化商贸旅游区、荔湾区十三行传统商贸业转型升级区、白云区大型现代商业中心、黄埔区临港商务区。加快中心城区产业“退二进三”和“腾笼换鸟”步伐，积极盘活旧厂房、旧工业用地，拓宽现代服务业发展空间。大力发展现代物流、现代商贸、金融保险、商务会展、文化创意、电子商务等现代服务业及总部经济，着力提升传统服务业发展水平。出台文化产业振兴规划及配套政策措施，促进文化创意产业加快发展。积极推进服务业综合改革试点，用好用足CEPA及其补充协议的各项政策，加快引进港澳服务业，推动服务业向集聚化、高端化和国际化发展，打造“广州服务”的品牌，整体提升服务经济的发展水平。中心城区外围以中新知识城、南沙新城、科学城、大学城、国际生物岛以及现代化空港、海港为依托，加快发展以知识经济为导向，以先进制造业和高新技术产业为主体的产业集群，打造若干个绿色增长区。重点抓好大型装备产业基地、龙穴造船基地、飞机维修基地、大功率机车检修基地、南沙核电产业园区等载体建设，引导和促进高端制造业集聚发展。加强与央企及国内外大型企业的战略合作，抓紧新能源汽车和三个年产百万辆轿车基地等重大产业项目筹划和申报工作，推动自主品牌乘用车项目建成投产。积极推进一批重大产业项目纳入全省“500强”建设规划。加大技改投入，加快优势传统产业和老工业企业技术改造，壮大制造业发展实力。大力推进“双转移”，确保实现省下达的年度考核目标。促进高新技术产业集聚发展，加快推进国家数字家庭应用示范产业基地、中国移动南方研发基地、中金数据中心华南基地、华南新药创制中心、广州（花都）光电子产业基地等重大项目建设。重点抓好计算机软件、集成电路、新型平板显示、数字电视与数字家庭、生物医药等高新技术产业发展，着力培育产值规模超千亿元、在国内外具有影响力的大型高新技术企业集团。

　　着眼长远加快培育发展战略性新兴产业。以抢占未来产业发展战略制高点为取向，立足我市产业特色和现实基础，研究制定我市战略性新兴产业发展规划和扶持政策。重点培育发展新能源、节能环保、电动汽车、新材料、新医药、海洋生物、软件和信息服务、新一代宽带无线移动通信、物联网与下一代互联网、半导体照明等新兴产业，力争将我市战略性新兴产业发展纳入国家和省的总体部署，使我市成为国家实施战略性新兴产业的重要基地，在新一轮产业革命中争取主动、把握先机。

　　充分发挥自主创新在转变经济发展方式中的核心作用。积极推进创新型城市建设，出台《广州国家创新型城市规划纲要》和《广州市提升自主创新能力行动计划》，加大财政对自主创新的支持力度，力争今年全社会研发经费占GDP比重达到2.4％。强化企业自主创新主体作用，吸引和支持创新要素、创新资源向企业集聚，加快形成产学研联盟。加强科技创新平台建设，集中力量攻克核心技术，力争形成一批自主创新的技术和产品标准。加快发展创业风险投资事业，为创新成果转化提供资金支持。大力推进知识产权战略，促进知识产权与经济发展、产业提升和自主创新相融合，提高知识产权资本化和产业化水平。加快广州高新区国家创新型科技园区建设。加强科技创新人才队伍建设。推动经济发展从要素驱动向创新驱动转变，努力实现从“广州制造”向“广州创造”的新跨越。

　　推动生产方式和生活方式向低碳型和循环型转变。加快建设低碳产业园区，推进广州开发区国家循环经济试点园区建设，启动千家企业清洁生产行动，大力开展节能、节水、节材和节地活动，促进资源的综合利用。全面落实节能减排工作责任制，确保实现“十一五”节能减排目标任务。大力推广应用低碳技术和产品，积极申报国家“十城万盏”半导体照明应用示范城市和国家节能与新能源汽车示范推广试点城市。创新路灯节能改造模式，大力推进全市路灯节能改造。出台鼓励支持低碳生产生活和消费的政策措施，加大宣传推广力度，培养全民节约意识和低碳意识，促进资源节约型和环境友好型社会建设。

　　（三）促进内外需协调发展，巩固持续向好的经济形势。

　　努力保持投资稳定合理增长。认真贯彻落实省委、省政府关于实施扩大内需战略的决定和扩大民间投资的政策，以重点项目建设带动和促进社会投资。今年全市89个重点项目计划安排总投资775.5亿元，预期带动全社会固定资产投资近3000亿元。把投资与迎亚运、强基础、调结构、保民生结合起来，进一步优化投资结构，改善投资服务，扩大投资需求。重点投向亚运场馆及配套设施、城市基础设施、环境建设、重大产业项目，以及社会民生和“三农”等工程项目。深化投资体制改革，加强重点投资项目责任管理，坚决完成省下达给我市的重点项目投资任务，并抓紧策划和储备一批新的项目，努力形成新的经济增长点。

　　积极扩大城乡居民消费需求。贯彻落实国家和省鼓励消费的战略，不断完善就业和社会保障制度，积极调控市场价格，保持价格总水平基本平稳，多渠道增加城乡居民收入，稳定居民消费预期，增强居民消费能力。开展“千年商都消费促进年”系列活动，进一步完善商业网点规划，增强城市核心商业街区的消费服务功能。打好“亚运牌”，充分发挥筹办亚运对经济的促进作用，开发更多的旅游产品，推动亚运文化旅游休闲消费，吸引海内外游客来穗观光消费。扩大家电、汽车、住房消费需求。实施“农村消费升级行动计划”，开拓农村消费市场。着力培育新型消费模式，促进信贷消费，支持发展节庆消费、租赁消费和网络消费。把握武广高铁开通带来的新商机，继续开展“广货北上”等商贸促销活动，努力扩大内销市场，增强消费对经济增长的拉动作用。

　　促进外经贸稳定增长。认真落实各项外经贸扶持政策，积极实施市场多元化战略，抓住中国-东盟自由贸易协定正式实施的重大机遇，引导企业调整出口目标市场，帮助企业开拓新兴市场。优化外贸进出口结构，推动汽车、船舶、机电和电子信息等高端产品出口，鼓励企业引进高端技术装备、关键零部件和重要能源资源及原材料。进一步优化投资环境，提高引资质量，争取更多大型企业落户广州。积极推进“中国服务外包示范城市”建设，组织企业承接国际服务外包，吸引国内外知名服务外包企业投资广州。鼓励和引导外资企业和加工贸易创建内销品牌，扩大内销比重。支持企业“走出去”，拓展发展空间，培育国际品牌和跨国经营企业。

　　扶持中小企业和民营经济加快发展。出台《进一步促进民营经济和中小企业发展的若干意见》，积极营造促进民营经济发展和推动民间投资的良好环境。完善融资担保体系建设，创新民营和中小企业融资模式，努力解决融资难问题。支持民营和中小企业投资公用事业、社会事业以及新兴产业和现代服务业，参与国有企业股份制改革。实施民营和中小企业成长培育工程，引导有条件的中小企业及民营企业建立现代企业制度，支持前景好、竞争力强的民营企业通过相互参股、收购兼并、外资嫁接等多种途径做大做强。支持广州民营科技园扩区，规划建设民营科技企业创新基地、节能和新能源产业基地，引导民营企业和中小企业集群化发展，不断增强广州经济的活力。

　　（四）切实办好改善民生十件实事，促进基本公共服务均等化。

　　一是确保全市新增就业岗位20万个。加快创建充分就业社区，推进省市共建广州就业实训基地建设，完善促进就业的长效机制。投入5.4亿元，加大对就业困难人员和“零就业家庭”的就业援助。大力开发公益性就业岗位，鼓励和支持劳动者自主创业和自谋职业。以高校毕业生为重点，加强就业指导和服务。落实税收优惠政策，推动以创业带动就业。确保实现城镇登记失业人员就业率70％以上，登记在册的“零就业家庭”1人以上就业率100％和城镇登记失业率控制在3.5％以内。

　　二是继续扩大社会保险覆盖面并提高保险水平。年底前将76万应参保农村居民纳入新型农村社会养老保险体系，使农村养老参保覆盖面扩大到70％。安排社保资金30.89亿元，进一步提高城镇企业退休职工、农转居人员、被征地农民和农村居民养老保险待遇水平。加快实现医疗保险市级统筹，建立基本药物制度，最大限度地减轻患大病、重病参保人医药费用负担。巩固和发展新型农村合作医疗制度，筹资标准提高到年人均260元以上，各级财政的补助标准提高到年人均 210元以上，提高参合农民医疗费报销比例，开展参合农民门诊统筹。

　　三是进一步提高社会福利水平。建立低保标准正常增长机制，完善城乡一体化低收入困难家庭认证和收入核算制度。提高抚恤补助标准，健全各类重点优抚对象医疗补助制度，确保享受抚恤补助的优抚对象100％纳入城乡医疗保障范畴。全面落实农村“五保”供养政策，进一步加强孤儿救助。完善养老体系建设，大力推广到户式和一站式的社区综合居家养老服务。全年新增养老床位4000张，实现每百名老人平均拥有床位数2.5张。进一步扩大老年人长寿金发放范围。落实市政府与中残联签订的协议，构建具有广州特色的残疾人社会保障和社会服务体系，在全社会形成扶残助残的良好风尚。

　　四是提升医疗卫生服务水平。完成市第八医院、市疾控中心和128所社区卫生服务中心、190个社区卫生服务站建设，实现城区社区卫生服务全覆盖。健全农村三级医疗卫生服务体系，推进镇卫生院标准化建设，扩大村卫生站看病减免收费试点。深化医药卫生体制改革，加快建立健全覆盖城乡居民的基本医疗卫生制度。全面实施重大公共卫生服务项目，免费为全市15万儿童补种乙肝疫苗，为14万农村妇女进行“两癌”检查，为2000名贫困患者施行白内障复明手术，为农村孕产妇提供住院分娩补助。

　　五是完成西江引水工程，让更多的市民喝上放心、安全、优质的水。加快推进西江引水工程建设，确保亚运会前实现通水，全面置换我市西北部地区水源，使西村、江村、石门等三间水厂水质达到新的国家《生活饮用水卫生标准》和《饮用净水水质标准》，惠及越秀、荔湾、白云等区以及天河区部分区域约600万市民。

　　六是完成100所义务教育学校规范化建设。按照市、区（县级市）1：1的比例，落实专项资金，优先保障用地，开辟绿色通道，全力推进义务教育规范化学校建设，确保年内全市规范化学校比例提高10个百分点，让更多的市民分享规范、优质的教育资源，促进义务教育均衡发展。

　　七是加速推进保障性住房建设。贯彻落实国家和省关于加大住房保障力度、加快保障性住房建设的精神，加快完善我市多层次住房保障供应体系。将原定于2012年底前解决7.72万户低收入住房困难家庭住房问题的目标，提前到2011年底前实现。在抓紧推进在建的221.27万平方米保障性住房建设的基础上，计划投入45亿元，新开工建设保障性住房4万套，建筑面积300万平方米，并储备3平方公里保障性住房建设用地。调整廉租房申请标准，将申请人年均可支配收入由7680元调整到9600元，提高住房保障普惠度。加快中低价位、中小户型普通商品住房建设，推出限价商品房用地。实行经济租赁住房制度，通过租售并举的方式，解决“夹心层”群体的住房困难问题。

　　八是进一步改善市民出行条件。加大道路交通建设力度，新建和续建道路188公里，建设44座人行天桥及一批步行连廊，提高车辆和行人通行效率。完善公共交通体系，及时调整和优化公交线网，完善快速公交（BRT）运营管理，推动公交地铁接驳换乘。更新1474辆老旧公交车，改造7800辆出租小汽车，改善车容车貌，提高服务质量。不断优化道路交通组织，严格占道施工管理，大力整治交通黑点，努力解决部分地区存在的交通拥堵问题。

　　九是推进全民健身设施建设。实施“融体于绿”工程，全年投入不少于2000万元，带动社会力量建设500处健身路径、球类运动、休闲活动等社区体育设施，形成遍布城乡、各具特色的体育健身设施网络。年内基本建成总长480公里、以自行车和步行为主要交通方式，集文化休闲、旅游观光和生态保护于一体的“绿道”走廊。加大公共体育场馆开放力度，使天河体育中心等亚运场馆成为市民健身活动重要场所。

　　十是推进百项政府服务网上办公工程。围绕市民和企业需求，以建立工商、社保、人力资源、民政、公安、卫生、国土、规划、税务、计生、质监和食品药品监督等网上办公与服务为突破口，推进市民网上服务和企业办事大厅建设，年内开设100项面向市民和企业的网上服务与办公事项，努力为广大市民和企业提供高效、便捷的服务。

　　（五）深化重点领域和关键环节改革攻坚，不断完善促进科学发展的体制机制。

　　深化行政管理体制改革。全面完成市、区（县级市）两级政府机构改革，理顺职能关系，促进大部门体制的磨合和有机衔接。推进简政放权改革，完善市对区（县级市）财政体制，促进财力与事权相匹配，增强基层政府履行职责和提供公共服务的能力。深化行政执法体制改革，加大镇（街）综合执法力度，强化行政执法督察机制。深化事业单位人事制度改革，完善有关配套政策，促进政事分开、事企分开。进一步深化行政审批制度改革，创新行政审批方式，提高办事效率。支持广州开发区实施行政事业性收费综合改革试点。

　　加快推进经济领域改革。推动国有企业改革向纵深发展，加快完善国有企业法人治理机制，落实企业投资决策权，建立科学的企业考核评估体系，健全企业领导人激励和约束机制，着力解决国企深层次矛盾和问题。加快国有企业公司制、股份制改造，加强资本运作，打造一批竞争力强的大型企业集团。继续深化广州银行和广州农村商业银行的改革重组，引进战略投资者，加快上市步伐。进一步深化城市建设投融资体制改革，促进七大投资集团在城市建设中发挥重要作用。加快建设国家级区域性产权交易市场。争取国家和省有关部门的支持，探索设立区域性柜台交易市场。积极申请恢复设立广州期货交易所，提升珠三角地区大宗商品交易的国际市场定价能力和定价水平。

　　加快推进文化体制改革。着力解决影响和制约文化科学发展的深层次矛盾和问题，巩固和发展经营性文化事业单位和文艺院团转企改制成果，打造合格的市场主体。深化新闻媒体宣传与经营业务“两分开”和制播分离改革，推进媒体经营性文化资产重组，培育一批实力雄厚、具有较强竞争力和影响力的领军企业。加大区（县级市）广播电视和有线电视网络资源整合力度，全面推进区（县级市）文化事业单位改革。

　　积极创新社会管理体制。加强对社会组织的培育和分类指导，重点扶持发展非营利性社会服务机构。扩大政府购买服务范围，提高社会公共服务的质量和效益。推动社会管理重心下移，创新街道社区管理服务模式，开展整合街道机构和人员试点工作，提高社区社会管理和公共服务水平。切实加强村（居）委会建设，促进城乡基层群众自治。建立具有广州特色的社会工作人才体系，探索在公益类事业单位、街道、社区以及非营利性社会组织中设置相应社会工作专业岗位，加快社会工作专业化和职业化进程。

　　（六）继续抓好“三农”工作，促进城乡一体化发展。

　　进一步加大强农惠农力度。深入贯彻落实市委、市政府《关于加快形成城乡经济社会发展一体化新格局的实施意见》，加快统筹城乡发展。积极调整财政支出结构，保证各级财政对农业投入增幅高于财政经常性收入增幅。加大对农村基础设施建设和社会事业发展的投入。整合支农资金，提高涉农资金使用效益。落实农业各项财政补贴和减免涉农税收规费等优惠政策，切实改善农业生产条件和减轻农民负担。

　　加强农业现代化建设。围绕构建都市型现代农业体系，进一步优化农业产业结构。以规划建设现代农业园区为重要载体，继续抓好标准化农田建设，推广设施农业建设。大力发展优质蔬菜、水产、花卉和特色果品产业，推进发展观光休闲农业和科技农业等新兴业态。抓好农业科技创新和推广能力建设，积极实施农产品品牌战略，大力发展农产品加工业，提高农产品附加值。做大做强农业龙头企业，重点推进广州花之都现代花卉产业基地、南沙都市型现代农业产业园和从化万亩鲜切花生产与旅游综合示范区建设，努力打造成为国家级现代农业园区。加强农产品市场和流通体系建设，提高农产品质量安全监管水平。

　　加快推进城镇化进程。着眼从根本上解决“三农”问题，以推进城镇化为抓手，继续完善中心镇基础设施建设，加快引导一批符合农村地区发展要求的“双转移”产业项目向中心镇及周边工业园区有序转移，增强中心镇人口和产业集聚能力。完善城镇落户政策，帮助符合条件的农业转移人口向城镇转移就业落户。加强社会主义新农村建设的规划引导，加快城市基础设施向农村地区延伸和覆盖。集中资金，选定一批村镇进行公共配套设施建设和村容村貌、危破房改造。制定农村路灯建设计划，逐步改变农村地区长期没有路灯的状况。加快农村社区建设，加强镇一级城市建设与管理机构建设，不断提高农村地区管理水平，促进农村人居环境持续改善。

　　多管齐下拓宽农民增收渠道。进一步完善城乡一体的职业技能培训和就业服务体系，积极帮助农村富余劳动力实现创业和就业。加快农民专业合作社发展，提高农民组织化程度。因地制宜发展农村家庭工业、社区服务业和乡村旅游业，提高农民从事二、三产业比例，促进农民增收。加快制定农村土地流转政策，建立健全农村土地承包经营权流转市场，积极引入社会资本参与农村土地承包经营权流转。推进土地入股等新型流转方式，形成农民、企业、集体三方共赢机制，增加农民财产性收入。深入推进农村改革和体制创新，为农民增收提供制度保障。

　　（七）全力推进《规划纲要》贯彻落实，加快国家中心城市建设。

　　全面贯彻落实《规划纲要》实施细则。按照实施细则提出的率先建立“十大体系”的安排，建立专项督办和评估考核机制，加强检查督促，落实工作责任，抓紧组织实施。尤其要着眼强化广州国际商贸中心、国际航运中心、知识创新中心和金融中心功能，编制相关专项规划，推进重大配套项目建设，推动广州实现新发展。

　　积极推进创新发展试验区建设。抓紧推进中新知识城建设，努力把知识城建设成为引领广州、广东乃至中国产业高端发展尤其是知识型经济发展的新引擎，汇聚全球精英人才的人才集聚高地，国际一流水平的生态宜居新城。抓住《规划纲要》明确规划建设南沙新区的重大机遇，充分利用南沙的优越条件，参照国家设立综合改革试验区的模式，积极争取在CEPA框架下，设立南沙穗港澳合作示范区，与香港在金融、贸易、投资、航运、中介、教育和科研等领域合作上开展先行先试。抓紧实施增城统筹城乡发展综合配套改革试点，探索县域经济科学发展新模式。抓住增城工业园区升格为国家级经济技术开发区的契机，打造新的经济增长极。

　　加快推动广佛同城化和广佛肇经济圈建设。落实广佛同城化建设和广佛肇经济圈建设合作框架协议，进一步完善合作机制，切实做好相关专业规划的衔接协调，落实既定的重点合作项目。重点加强与佛山的城际分工合作，加快推进城际轨道等交通基础设施无缝对接，促进教育文化、医疗卫生、科技信息等联动发展，实现区域资源共建共享。深化与深莞惠、珠中江经济圈的合作，促进珠三角一体化。积极探索穗港澳合作新方式，拓展合作空间，提升合作层次，实现互利共赢，努力在珠三角一体化进程中发挥好示范带头作用。

　　科学做好“十二五”规划编制工作。以加快建设国家中心城市、全面提升科学发展实力为主线，组织开展“十二五”规划前期课题研究和编制工作，为广州新一轮大发展谋篇布局。

　　（八）进一步加强社会事业建设，全面提升社会文明程度。

　　加强精神文明建设。坚持以建设社会主义核心价值体系为根本，切实加强公民道德建设。推动创建全国文明城市工作常态化、规范化、精细化，着力深化群众性精神文明创建活动，积极倡导文明健康的生活方式，大力营造文明和谐的社会风尚，进一步提高市民素质和城市文明程度。

　　大力繁荣文化、教育和体育事业。发展哲学社会科学，繁荣新闻出版、广播影视、文学艺术。加强文化精品创作，增加公共文化产品，丰富人民群众的精神文化生活。扎实推进各项重点文化设施建设，继续完善基层文化服务网络，打造全省一流、全国领先的公共文化服务体系。探索建立长效机制，提高公共文化服务水平。精心办好第九届中国艺术节等文化品牌活动。完善文化市场管理和“扫黄打非”体制机制，加强网络文化建设和管理。重视文物和非物质文化遗产的发掘、保护和传承。加强科普工作，提高市民科学素养。探索普通高中招生制度改革，促进高中教育整体优质发展。将农村义务教育经费全部纳入政府公共财政保障范围，提高农村中小学生人均公用经费标准。全面实施城乡贫困家庭子女高中阶段免费教育。加快中职学校新一轮布局调整和基础能力建设，推动市属高校规模、质量、结构、效益协同发展。依法落实教师待遇，提高在职、退休教师的收入水平。加快发展体育事业，大力开展健康教育和健康促进行动。提高竞技体育水平，争取我市运动员在广州亚运会上再创好成绩。

　　促进社会各项事业新进步。完善和落实人口政策，稳定低生育水平，统筹解决人口问题，促进人的全面发展。加强应对人口老龄化政策措施研究，建立健全养老社会服务体系，发展老龄事业。进一步深化与世界城市和地方政府联合组织（UCLG）的交流合作，拓展友城关系，提升对外开放水平。做好对台和侨务工作。加强防灾减灾基础设施建设，提高气象、地质、防灾减灾工作水平。继续加强对汶川威州镇的帮扶援助，促进灾区的新发展。全面推进“规划到户、责任到人”扶贫开发工作，坚决完成省下达给我市的任务。深入开展构建“法治广州”活动，加强市民法制教育。建立和完善人民调解、司法调解、行政调解、仲裁调解相衔接的工作机制，进一步扩大人民调解的覆盖面和影响力。加强法律服务和援助，最大限度地满足建设法治社会的法律需求和困难群体的法律需求。充分发挥工会、共青团、妇联等人民团体和社会组织参与社会管理和公共服务的重要作用。以开展亚运志愿服务为契机，推动志愿服务事业新发展。认真落实党的民族政策和宗教工作基本方针。开展民族团结创建活动，进一步改善宗教服务，规范宗教事务管理。做好第六次全国人口普查工作和第二次全国经济普查后续工作。全面落实优抚政策，妥善安置转业、退伍军人和移交地方的军队离退休干部，加强民兵、预备役部队建设和海防、人防及打击走私工作，提高双拥共建水平。

　　（九）切实加强政府自身建设，适应经济社会发展转型的新要求。

　　努力改进政府工作。针对经济社会发展转型和施政环境的新情况、新变化，加快转变政府职能，更加重视公共服务和社会管理，提高政府的执行力和公信力，加快建设服务型政府。加强对公务员的教育、培训、考核和管理监督，强化公仆意识，落实行政问责制度，做到有错必纠，有责必问，问责必严。进一步转变工作作风，密切联系群众，坚持问政于民、问需于民、问计于民，高度重视和认真回应群众诉求。完善政务服务系统建设和政府信息公开机制，推进重大决策公开，提高政府工作透明度，保障行政权力公开规范高效运行。协调处理好公共关系，建立媒体、公众、政府的良性互动机制，营造良好的舆论氛围。

　　全面推进依法行政。认真执行人大及其常委会的决议、决定，接受人大及其常委会的法律监督、工作监督和人民政协的民主监督，定期报告重大工作事项，切实做好人大代表议案、建议、批评和意见及政协提案办理工作。完善市民听证制度和网络问政制度，制定和推行重大行政决策程序规定，健全行政决策合法性审查机制，促进科学民主决策。发挥公职律师和政府法律顾问的作用。主动听取各民主党派、工商联、各人民团体及社会各界人士的意见建议。做好政府规章制定工作，规范行政执法和行政行为，创新行政复议工作机制，促进政府工作法制化。

　　大力加强廉政建设。加强以建立健全预防和惩治腐败体系为重点的反腐倡廉建设，落实廉政建设责任制，推进反腐倡廉制度建设，完善公共权力运行制约和监督机制，从源头上预防和治理腐败。坚持艰苦奋斗、勤俭节约。充分发挥监察、财政和审计等监督作用，规范预算管理，推进绩效审计，加强对政府重点投资项目和亚运建设项目的跟踪审计，切实做到节俭办亚运、廉洁办亚运。加大查办腐败案件力度，依法严惩各类违法违纪行为，坚决纠正各种不正之风。

　　各位代表，做好今年经济社会各项工作，使命光荣，任务艰巨。让我们更加紧密地团结在以胡锦涛同志为总书记的党中央周围，在市委的领导下，奋发开拓，扎实工作，为促进广州经济社会又好又快发展，为创造广州人民幸福安康、和谐美好的新生活而努力奋斗！
